# Prevalence and causes of visual impairment amongst hearing impaired school-going children in sub-Saharan Africa: a scoping review

**DOI:** 10.4314/ahs.v22i4.24

**Published:** 2022-12

**Authors:** Michael Agyemang Kwarteng, Khathutshelo Percy Mashige, Samuel Kyei, Daniel Sunkwa Quarcoo Dogbe, Pirindhavellie Govender-Poonsamy

**Affiliations:** 1 Department of Optometry, Faculty of Science and Engineering, Bindura University of Science Education, Bindura, Zimbabwe; 2 Discipline of Optometry, School of Health Sciences, University of KwaZulu-Natal, Durban, South Africa; 3 Department of Optometry and Vision Science, School of Allied Health Sciences, College of Health and Allied Sciences, University of Cape Coast, Cape Coast, Ghana; 4 Department of Special Education, Faculty of Education, University of Education, Winneba, Ghana

**Keywords:** Visual impairment, prevalence, Africa, hearing impairment, ocular morbidity

## Abstract

**Background:**

Learners living with hearing impairment are at a higher risk of visual impairment.

**Purpose:**

To summarise relevant literature investigating the prevalence and causes of visual impairment amongst learners living with hearing impairment in sub-Saharan Africa.

**Methods:**

A search of nine databases and the reference lists of retrieved studies were conducted using the standard methodology for scoping reviews as described in the PRISMA statement. The databases were MEDLINE, PubMed, EMBASE, the Cochrane Library, Global Health, OVID, Google Scholar, Web of Science, and Science Direct. A scoping review of articles published in the English language from 2000 to 2020 was conducted while considering the study design, sub-Saharan Africa, and school for the deaf. Descriptive statistics was used to analyse the data.

**Results:**

The initial search retrieved eight studies, seven of which met the set inclusion criteria. All seven studies included employed a cross-sectional design. The prevalence of visual impairment ranged from 2.2–34.6% with the major cause being uncorrected refractive error (7.9–73.26%). The most common type of refractive error was myopia (42.2%) followed by hyperopia (28.6%) and astigmatism (28.6%).

**Conclusion:**

This review has demonstrated that there is a paucity of high-quality and well-designed studies that have investigated the prevalence and causes of visual impairment amongst hearing-impaired children in sub-Saharan Africa suggesting the need for further research in this area.

## Introduction

The most significant causes of visual impairment amongst children is uncorrected refractive error accounting for 47 – 92% of all ocular morbidities (any eye condition regardless of resultant vision loss).^1^ In sub-Saharan Africa (SSA), studies have reported that ocular morbidities amongst children are high due to low access to eye care facilities and poor uptake of eye care services.^2^–^4^ The prevalence of uncorrected refractive error in Africa has been reported to range from 1.4 – 8.5% among children. 1–10 Children with hearing impairment in particular rely primarily on their visual-perceptual cues for their activities of daily living.^11^,^12^ Therefore, any impairment of their vision can greatly affect their quality of life.

Reports suggest that the prevalence of ocular morbidities and refractive error amongst children with hearing impairment is 5 - 90.1% and 7.2 - 62.65%, respectively.^11^–^28^ Most of the causes of the reported visual impairment are avoidable. Also, studies have reported that children with hearing impairment are at a higher risk of visual impairment than children with no impairment.^11^,^12^ In sub-Saharan Africa, not much attention has been given to visual impairment in children with hearing impairment. The aetiology of the significant causes of childhood visual impairment and hearing impairment in Sub-Saharan Africa is associated with measles and rubella .^17^

There still exists the lack of scientifically rigorous studies that have investigated the prevalence and causes of visual impairment amongst learners living with hearing impairment in sub-Saharan Africa. Therefore, the aim of this scoping review is to evaluate published scientific research studies on the prevalence and causes of visual impairment in children living with hearing impairment in terms of their visual acuity status, causes, interventions provided/suggested and limitations of the studies.

## Methods

This study was conducted using the recommended search strategy as described in the Preferred Reporting Items for Systematic Reviews and Meta-Analyses (PRISMA) statement.^29^ The objectives of this study were formulated based on the Population, Intervention, Comparison, Outcome (PICO) tool, which is the recommended tool for the synthesis of quantitative studies.^30^ A preliminary research and validation of the research topic were done by identifying relevant works in literature and ensuring that the question has not been addressed. The preliminary research was done using Google Scholar and PubMed using the terms visual impairment, hearing impairment, and Africa.

### Eligibility criteria

Inclusion criteria were based on the PICO tool, articles published in English, date of publication (2000–2020), study design, sub-Saharan Africa and school for the deaf. Unrelated articles, abstract-only papers, duplicates, related studies outside the study setting were excluded.

### Information Sources and Data Search

Nine databases were used in this study that met the guidelines of assessment of multiple systematic reviews (AMSTAR). 31 These databases were MEDLINE, PubMed, EMBASE, the Cochrane Library, Global Health, OVID, Google Scholar, Web of Science (SCI, SSCI, A&HCI, CPCI-S, and CPCI-SSH), and Science Direct. Prevalence and causes of visual impairment in school children with hearing impairment and other keywords were used in the data search with the help of an information scientist. Also, additional keywords from retrieved data were used to improve the search as recommended by Tawfik et al.^32^

### Study Selection

The initial search yielded a total of eight records, but seven studies met the inclusion criteria. The study which was excluded was conducted in special schools among participants with intellectual, visual, hearing, physical and multiple disabilities with no distinct classification of visual impairment among the different groups.

## Results

The analysis was based on the seven studies that met the inclusion criteria (see Table 1). All the studies employed a cross-sectional study design and all except one were conducted in Nigeria. Participants' age ranged from 5–39 years and the number of participants ranged from 86–620 with five studies reporting more males than females. The prevalence of visual impairment ranged from 2.2–34.6% with the major cause reported as uncorrected refractive error (7.9–73.26%). The major type of refractive error was myopia (3 studies), hyperopia (2 studies), and astigmatism (2 study). The prevalence of ocular morbidities ranged from 20.9–73.26% and refractive error was the leading cause.

**Table 1:** Summary and comparison of study findings according to authors

Author(s)	Year	Country	Age range (years)	Number	Prevalence of VI (%)	Causes of VI	Prevalence of RE (%)	Major cause of RE	Prevalence of OM (%)	Common cause of OM
Osiayuwu Ebeigbe^33^	2009*	Nigeria	6–16	86	-	RE	73.26	Myopia	73.26	RE, strabismus
Onakpoya and Omotoye^34^	2008	Nigeria	6–25	156	4.5	RE	18.6	Myopia	34.0	RE, retinal changes
Abah et al^35^	2011*	Nigeria	5–38	620	2.2	RE	7.9	Hyperopia	20.9	RE, allergic conjunctivitis
Ovenseri- Ogbomo et al^39^	2011	Ghana	9–27	243	7.3	RE	31.9	Astigmatism	21.4	RE, retinal
Omolase et al^36^	2011	Nigeria	5–23	160	-	RE	38.1	Myopia	26.25	RE, glaucoma
Majekodunmi et al^37^	2016	Nigeria	11–39	335	34.6	RE	56.1	Hyperopia	56.1	RE, allergic conjunctivitis
Abikoye et al^38^	2020*	Nigeria	7–19	109	19	RE	11	Astigmatism	50.5	Allergic conjunctivitis, RE

OM = Ocular Morbidity; RE = Refractive error; VI = Visual impairment

* year of publication

No * = year of study

In Nigeria, Osiayuwu and Ebeigbe^33^ reported in 2009 that their participants comprised 39.5% males, 60.5% females, and 73.3% had visual disorders. They reported that the proportion of visual problems decreased with increasing age. However, the limitations of the study were the lack of categorizations of visual impairment itself and learners according to their visual impairment. In 2010, Onakpoya and Omotoye^34^ reported 48.1% males and 51.9% females' representation in their study in Nigeria. The authors found that 4.5% of their participants had visual impairment and 34% had ocular morbidity. Onakpoya and Omotoye study did not differentiate between the various kinds of hearing impairment and deafness (mild, moderate, severe) among the learners. Abah et al.,^35^ in 2011 reported that 20.9% of their learners had visual disorders and 2.2% had visual impairment. Their participants included 61.35% male and 38.7% female learners. The authors did not classify the learners according to the type of hearing impairment, albeit the inclusion of an otorhinolaryngologist in the study's team. A Nigerian study in 2012 by Omolase et al.,^36^ found that their study participants comprised of 56.9% male and 43.1% female learners, with 20.9% having visual problems and 2.2% visual impairment. The limitation of Omolase et al study is that they classified visual impairment for the right and left eyes instead using the better-seeing eye. Another study conducted in Nigeria by Majekodunmi et al.,^37^ in 2018, reported that 56.1% of the learners had visual problems and 34.6% had visual impairment. The participants included 58.2% male and 41.8% female learners. Majekodunmi et al study did not differentiate between the various kinds of hearing impairment among the learners. Furthermore, Abikoye et al.,^38^ stated in 2020 that their participants were split evenly between 51.4% men and 48.6% females. The kinds of hearing impairment were not classified in the research.

In 2013, Ovenseri-Ogbommo et al.,^39^ reported a 7.3% prevalence of visual impairment among 58% male and 42% female learners in Ghana. Similar to the study by Omolase et al, the researchers reported visual acuities in the right and left eyes without considering the better-seeing for the classification of visual impairment.

## Discussion

This is the first literature review to report on the causes and prevalence of visual impairment amongst learners with hearing impairment in sub-Saharan Africa. The prevalence of ocular morbidities was high, with the leading cause being refractive error. Allergic conjunctivitis was the second most common cause of ocular morbidities. In sub-Saharan Africa, eye care delivery is unevenly distributed due to inadequate equipment and skilled personnel, with most of the secondary and tertiary eye care institutions located in major urban centres and national capitals.^40^ In SSA, only one-quarter of the countries meet the minimum eye care workforce expectation of one worker per 55,000 people.^40^ The inadequate distribution of the eye care workforce in SSA has resulted in the high prevalence of ocular morbidities. There are glaring deficiencies in visual function data among learners with hearing impairment due to the lack of studies in other parts of the continent since all of the studies were Nigerian-based except one. This is a major issue and attention should be given to visual function and ocular problems amongst learners with hearing impairment across Africa. The age of the learners ranged from5–39 years, which indicates the late presentation to schools and the pursuit for vocational education and training, which are available to learners with disabilities. This result is similar to the age range of learners in schools for the blind in Africa, particularly Ghana and Nigeria, due to the late start of school, inadequate support from family, and academic challenges which retard academic progress.^41^ In terms of sex distribution, five of the studies had more male than female learners, which possibly reflects the trend in Ghana and Nigeria where male learners are more likely to be in school than females due to culture and traditions. This trend is similar to reports among learners with hearing impairment in Asia^11^ and also learners with other disabilities in Africa^41^. In contrast, Osiayuwu and Ebeigbe^33^, and Onakpoya and Omotoye^34^ reported more females than males in their study. The reason for this variation could not be determined based on location since all the schools were located in the urban areas, and also ethnic variation since the difference was from the same state. However, female learners with disabilities encounter an inequitable primary and secondary education as the African society values education for the boy-child than the girl-child. 37,42

The prevalence of ocular morbidity was high (20.9–73.26), which can remain undetected and impact the learners' academic pursuits. The high prevalence can be reduced through periodic eye screening, better screening protocols amongst the learners, and awareness of eye health among caretakers such as parents and teachers since these learners rely primarily on visual cues. Furthermore, an increase in the workforce in eye care can reduce the prevalence of ocular problems among these learners. The common ocular disorder was refractive error which is consistent with other reviews among learners with hearing impairment.^11^,^43^ However, the prevalence of refractive error is high in this study compared to the reported studies in other continents.^11^,^43^ The lack of a rigorous eye care system at the primary and secondary level, centralised eye care personnel and cost of refractive error correction aids in Ghana^44^ and Nigeria^45^ might have resulted in the high prevalence. Also, poor level of eye care service utilization, which results from inadequate funds, late presentation and lack of an escort to health facilities among parents/guardians of these learners may contribute to the high prevalence.^34^,^38^,^46^ Eye health education and promotion will improve the uptake of eye care utilization, which will reduce the prevalence among learners with hearing impairment.

All the seven studies provided evidence for the type of refractive error. Studies by Osiayuwu and Ebeigbe^33^, Onakpoya and Omotoye^34^, and Omolase et al.,^36^ reported that myopia was the commonest type of refractive error. In contrast, Abah et al.,^35^ and Majekodunmi et al.,^37^ reported hyperopia as the commonest while Ovenseri-Ogbomo et al.,^37^ and Abikoye et al.,^38^ found astigmatism as the commonest. A possible reason would be the use of different definitions and classification of refractive errors by the authors. Ovenseri-Ogbomo et al.,^37^ and Abikoye et al.,^38^ defined myopia as spherical power equal to or greater than -0.50D, hyperopia as spherical power equal to or greater than +2.00D, and astigmatism equal to or greater than 0.50D, while Majekodunmi et al.,^37^ defined myopia as spherical power equal to or greater than -0.50D, hyperopia as spherical power equal to or greater than +0.50D, and astigmatism greater than 0.50D. The remaining four studies did not provide the definitions used in classifying the types of refractive errors. A global review by Hollingsworth et al.,^43^ reported hyperopia as the most common refractive error among children with hearing impairment followed by myopia and astigmatism. However, the study by Hollingsworth et al.,^42^ included just one study from sub-Saharan Africa.

A number of indicative findings and recommendations emerged from this study;

1 A recommended definition and classification of visual impairment and refractive error should be used in reporting research findings.

2 Researchers should classify the types of hearing impairment (mild, moderate, severe) and deaf among learners in the Schools for the Deaf. Knowing the specific impairment will inform stakeholders on the plight of the learners and their management.

3 Corresponding authors should be contacted to clarify any inconsistencies in their articles to avoid citing different prevalence rates.

4 There is a need for more research involving learners with hearing impairment across sub-Saharan Africa and classifying them according to their impairment to meet their visual needs. Learners with hearing and visual impairment would require special teaching and learning environment to improve their quality of life.

## Conclusion

This study reflects a high prevalence of avoidable ocular morbidities and visual impairment amongst learners with hearing impairment, with refractive error as the main cause. This information can provide the evidence to justify and inform planning, policies and management strategies amongst learners living with visual and hearing impairment in Ghana and Nigeria. This data also provides evidence from which the priorities and scope for further research amongst learners with hearing impairment in SSA can be planned.
